# The Genome of Blue-Capped Cordon-Bleu Uncovers Hidden Diversity of LTR Retrotransposons in Zebra Finch

**DOI:** 10.3390/genes10040301

**Published:** 2019-04-13

**Authors:** Jesper Boman, Carolina Frankl-Vilches, Michelly da Silva dos Santos, Edivaldo H. C. de Oliveira, Manfred Gahr, Alexander Suh

**Affiliations:** 1 Department of Evolutionary Biology, Evolutionary Biology Centre (EBC), Science for Life Laboratory, Uppsala University, SE-752 36 Uppsala, Sweden; 2 Department of Behavioral Neurobiology, Max Planck Institute for Ornithology, 82319 Seewiesen, Germany; frankl@orn.mpg.de (C.F.-V.); gahr@orn.mpg.de (M.G.); 3 Laboratório de Cultura de Tecidos e Citogenética, SAMAM, Instituto Evandro Chagas, Ananindeua, Pará, and Faculdade de Ciências Naturais (ICEN), Universidade Federal do Pará, Belém 66075-110, Brazil; michellyufpa@gmail.com (M.d.S.d.S.); ehco@ufpa.br (E.H.C.d.O.)

**Keywords:** transposable elements, transposons, LTR retrotransposons, ERV, genome, genome annotation, karyotype, estrildidae, zebra finch, *Uraeginthus cyanocephalus*

## Abstract

Avian genomes have perplexed researchers by being conservative in both size and rearrangements, while simultaneously holding the blueprints for a massive species radiation during the last 65 million years (My). Transposable elements (TEs) in bird genomes are relatively scarce but have been implicated as important hotspots for chromosomal inversions. In zebra finch (*Taeniopygia guttata*), long terminal repeat (LTR) retrotransposons have proliferated and are positively associated with chromosomal breakpoint regions. Here, we present the genome, karyotype and transposons of blue-capped cordon-bleu (*Uraeginthus cyanocephalus*), an African songbird that diverged from zebra finch at the root of estrildid finches 10 million years ago (Mya). This constitutes the third linked-read sequenced genome assembly and fourth in-depth curated TE library of any bird. Exploration of TE diversity on this brief evolutionary timescale constitutes a considerable increase in resolution for avian TE biology and allowed us to uncover 4.5 Mb more LTR retrotransposons in the zebra finch genome. In blue-capped cordon-bleu, we likewise observed a recent LTR accumulation indicating that this is a shared feature of Estrildidae. Curiously, we discovered 25 new endogenous retrovirus-like LTR retrotransposon families of which at least 21 are present in zebra finch but were previously undiscovered. This highlights the importance of studying close relatives of model organisms.

## 1. Introduction 

Birds are remarkable among vertebrates by having small genomes, a low variation (0.91–2.16 pg, 2.4-fold) in genome size and a low density of repetitive elements [1,2,3]. Small genome sizes of birds are typically explained as an adaption for flight, through association with high metabolic rate which in turn selects for small red blood cells capable of greater gas exchange per unit volume [4,5,6]. This view is consistent with the observation of smaller genomes in flighted versus flightless birds and more streamlined genomes of bats compared to other eutherians [4,7,8]. However, measurements of insertion and deletion rates suggest that birds with more transposable element (TE) accumulation also have more deletions, resulting in a higher net shrinking and therefore smaller genomes [3]. Larger genome sizes of flightless birds result from low deletion rates and accumulation of TEs, meaning that they have less genomic turnover overall [3]. This might indicate that genome size differences among extant birds do not necessarily reflect adaptation for flight, but instead lineage-specific differences in genome dynamism [3].

Birds are the most species-rich group of land vertebrates as a result of a massive radiation following the demise of other dinosaur fauna at the Cretaceous–Paleogene extinction event 65 Mya [9]. The putative association between TE accumulation and speciation that has been shown in, e.g., mammals [10] is an interesting prospect for avian TE biology. Transposons have for example been implicated as hotspots for chromosomal breakpoint regions [11,12,13], conceivably associating transposon accumulation with chromosomal inversions. Through recombination suppression, inversions may act as islands of genomic differentiation (e.g., [14]). Research has shown that the genome of the important model organism zebra finch has undergone many inversions on a short evolutionary timescale [15,16]. Zebra finch also has a recent accumulation of endogenous retrovirus (ERV)-like long terminal repeat (LTR) retrotransposons [17], which proliferate through a copy and paste mechanism [18]. Romanov et al. [16] found a positive correlation between LTR retrotransposons and genomic regions especially prone to chromosomal rearrangements, so-called evolutionary breakpoint regions. Moreover, intra-chromosomal rearrangements such as inversions are more frequent in the zebra finch’s family Estrildidae, than in other bird lineages [15]. 

To understand the dynamics of LTR proliferation in Estrildidae, we de-novo sequenced and karyotyped the genome of blue-capped cordon-bleu (*Uraeginthus cyanocephalus*) and performed an in-depth computational prediction and manual curation of TEs. Blue-capped cordon-bleu is an East African estrildid finch and famous for its rapid tap dancing display [19,20]. It belongs to a lineage that split from the Austro-Pacific zebra finch at the root of Estrildidae 10 Mya [15]. In-depth annotations of TEs consisting of both computational prediction and manual curation have so far only been presented for zebra finch, chicken (*Gallus gallus*) and collared flycatcher (*Ficedula albicollis*) [17,21,22]. Each genome curated has revealed a great diversity of new transposon families and subfamilies. Through rigorous manual curation, we discovered 25 new ERV-like retrotransposon families of which 21 are shared with zebra finch. Using repeats from collared flycatcher and blue-capped cordon-bleu, we find an additional 4.5 Mb of LTR elements (i.e., >10% increase in annotated bp) in the zebra finch genome assembly taeGut2, compared with using only previously curated bird repeats from Repbase. Furthermore, we show that blue-capped cordon-bleu has experienced a recent accumulation of LTR retrotransposons, which indicates that this is a shared feature of estrildid finches and likely important in shaping their genomic landscape.

## 2. Materials and Methods 

### 2.1. Sequencing, Genome Assembly and Karyotyping

**Figure 1:**
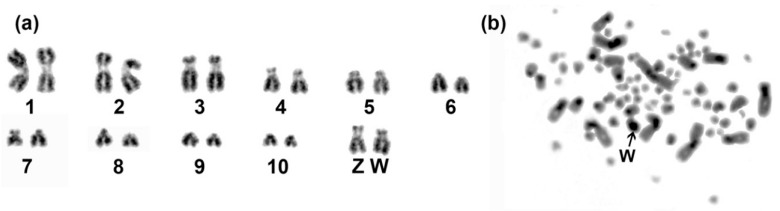
Karyotype of a female blue-capped cordon-bleu. The diploid (2n) chromosome number is 80. Giemsa staining of macrochromosomes showed that the sex chromosomes are approximately equal in size (**a**). C-banding revealed that the W chromosome is enriched in heterochromatin, compared to autosomes in which heterochromatin is restricted to putative centromeric regions (**b**). In panel (**a**), autosomes are numbered from largest to smallest, as proposed by the International System for Standardized Avian Karyotypes [57].

We sequenced the genome from heart and testis tissues of a male blue-capped cordon-bleu (*U. cy.*) bred at Max Planck Institute for Ornithology (Germany), Seewiesen animal facility, using the 10X Genomics Chromium linked-read system [23,24] and sequencing of 150-bp paired-end reads on an Illumina HiSeq X instrument, both conducted by SciLifeLab Stockholm (Sweden). Animal handling was carried out in accordance with the European Communities Council Directive 2010/63 EU and the legislation of the state of Upper Bavaria. We used a genome assembly from testis tissue for RepeatModeler prediction (see below), but decided to use an assembly from heart tissue for all analyses, to be more comparable with the somatic repeatomes of zebra finch, collared flycatcher and chicken, due to the recent hypothesis of a germline restricted chromosome being widespread among songbirds [25,26,27]. Hereafter, “the genome of blue-capped cordon-bleu” refers to the heart assembly. We generated “pseudohaploid” draft genome assemblies using Supernova 2.0 [23,24]. The Chromium system employs a unique barcoding of reads from the same input DNA molecule which potentially allows for the assembly of longer contigs and scaffolds than conventional short-read technologies [24]. We assessed the assembly quality using the assemblathon_stats.pl script [28] and investigated the gene set completeness using the aves_odb9 library in BUSCO2 [29] (Table 1). Karyotyping was performed on fibroblast cells from the embryos of both male and female blue-capped cordon-bleu using established protocols [30,31] with modifications described previously in Santos et al. [32] and Furo et al. [33] (Figure 1).

### 2.2. Computational and Manual Curation of Transposable Elements

Repetitive element consensus sequences were predicted de novo using RepeatModeler ver. 1.0.8 [34]. The predicted library of consensus sequences was masked with RepeatMasker ver. 4.0.7 using the *Aves* Repbase library [35]. Consensus sequences more than 5% diverged from previously annotated zebra finch repeat consensuses [17] were selected for manual curation. Using a custom script [22], the 20 best BLASTn ver. 2.6.0+ [36] hits of each consensus sequence along with 2-kb flanks were aligned using MAFFT ver. 7.310 [37]. For each repeat predicted by RepeatModeler, a new majority rule consensus sequence was made based on the aligned hits, either manually with an alignment viewer (Aliview [38] or BioEdit [39]) or using Advanced Consensus Maker [40]. At each site, the most abundant base was used as consensus, except for potential hypermutable CpG sites, which were curated as 5′-CG-3′. Target site duplication (TSD) patterns and the long terminal repeat (LTR) canonical 5′-TG…CA-3′ ends were used to identify and classify LTR retrotransposons into three groups [41]: endogenous retrovirus superfamily 1 (ERV1, 4 bp TSD), endogenous retrovirus superfamily K/2 (ERV2, 6 bp TSD) and endogenous retrovirus superfamily L/3 (ERV3, 5 bp TSD). The characteristic eight base pair motif [42], 5′-ATTCTRTG-3′, was used to identify the 3′ ends of CR1 LINEs. CR1 curation proceeded in 5′ direction as long as at least three BLASTn hits with high similarity were distinguishable in the alignment. 

Manually curated consensus sequences were queried against Repbase using CENSOR [43]. To date, a majority of avian repeats in Repbase are from chicken and zebra finch. SINE and LTR retrotransposons with considerable nucleotide similarity (>80%) across a majority of their lengths (>80%; for at least 80 bp) to a repeat in Repbase or to each other (checked manually), were classified as belonging to the same family. SINE and LTR retrotransposons with hits to Repbase that did not meet these criteria were classified as new families. The criteria used here are based on the TE family 80-80-80 rule cutoff proposed by Wicker et al. [44] in which two TEs belong to the same family if 80% of a novel TE is more than 80% identical for at least 80 bp of an already classified TE, in a BLAST search or similar against a repeat database. By the same classification scheme, a TE subfamily represents a subpopulation of an already identified TE family [44]. We classified novel TEs from the same species as belonging to separate subfamilies if their consensus sequences were less than 95% similar on the nucleotide level. Some blue-capped cordon-bleu consensus sequences were more than 95% similar to zebra finch repeats after manual curation (Table S1). We still consider these as separate subfamilies in our analyses. For all curated LTR retrotransposons that met our criteria for a novel family, we next searched a library of collared flycatcher LTR consensus sequences [22] using BLASTn (E-value = 0.01). We classified a blue-capped cordon-bleu LTR consensus sequence as belonging to a collared flycatcher LTR family if it had considerable nucleotide similarity across the majority of its sequence (see criteria above) to a collared flycatcher LTR consensus. CR1 elements were classified based on a PhyML ver. 3.0 [45,46] maximum likelihood (ML) phylogeny (GTR+G+I substitution model) of all CR1 subfamilies from blue-capped cordon-bleu, chicken, zebra finch and collared flycatcher. The library for the latter three is the same as in Suh et al. [22]. This and another phylogenetic tree of songbird repeats from the TE family TguERVL2_I were depicted using FigTree ver. 1.4.3 (Figures S1 and S2) [47]. TE subfamilies and families were named following previous conventions used in the zebra finch and collared flycatcher repeat annotations [17,22].

### 2.3. Data Analysis

**Figure 2:**
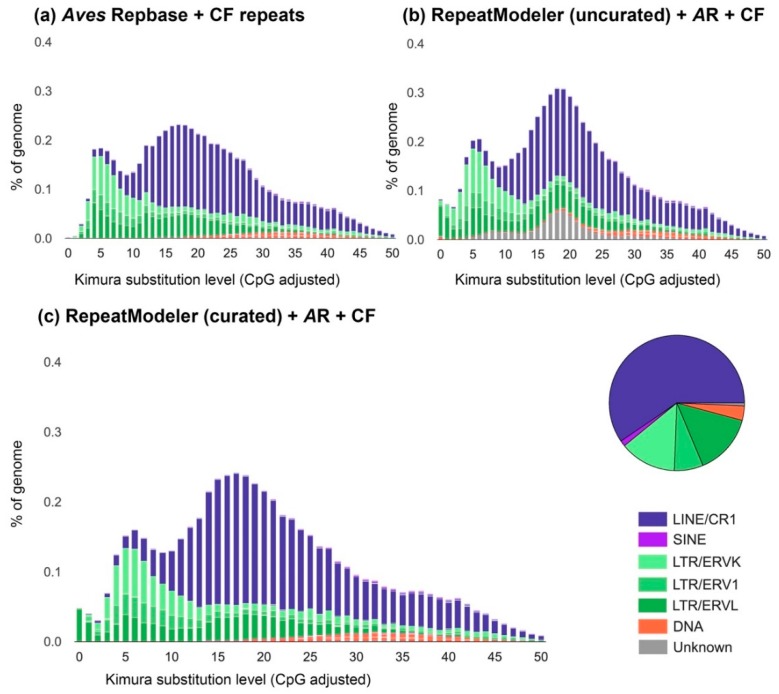
Comparison of transposable element landscapes for the genome of blue-capped cordon-bleu, representing different levels of effort in transposon annotation. Percentage of bp occupied in the genome is plotted against the Kimura 2-parameter (transitions/transversions) distance of each transposable element (TE) copy from its consensus. Panel (**a**) shows the landscape for when avian repeats available in Repbase (*Aves* Repbase, *A*R) and collared flycatcher (CF) repeats were used for masking the genome. Panel (**b**) is based on de-novo predicted repeats from Repeatmodeler, *A*R and CF repeats. Note the share of unknown (grey) repeats, a majority of which were identified as solo-long terminal repeats (LTRs) of endogenous retrovirus (ERV)-like retrotransposons when manually curated (**c**). The pie chart specifies the relative abundance of different TEs based on the .tbl file of the RepeatMasker output (Table 2), for the curated, final landscape (**c**).

**Figure 3:**
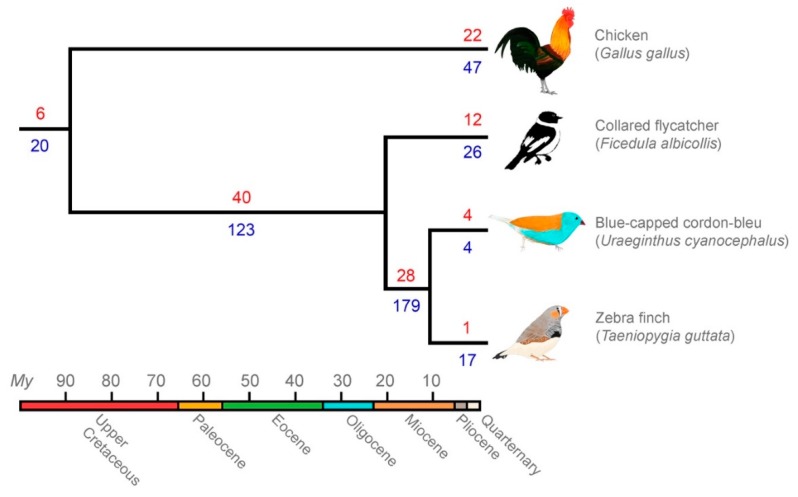
Analysis of LTR diversity along branches in the tree of birds with in-depth curated TE libraries. The number of LTR families and subfamilies on each branch are depicted in red (above branches) and blue (below branches), respectively. Most LTR retrotransposon families are shared between blue-capped cordon-bleu and zebra finch. The previously thoroughly investigated genome of zebra finch contains more lineage-specific TE subfamilies. A large diversity of LTR families and subfamilies are shared among the three songbirds compared with the relative sparse number of LTRs shared with chicken at the root of the tree. Node estimates are based on previously published timetrees [9,15,58].

**Figure 4:**
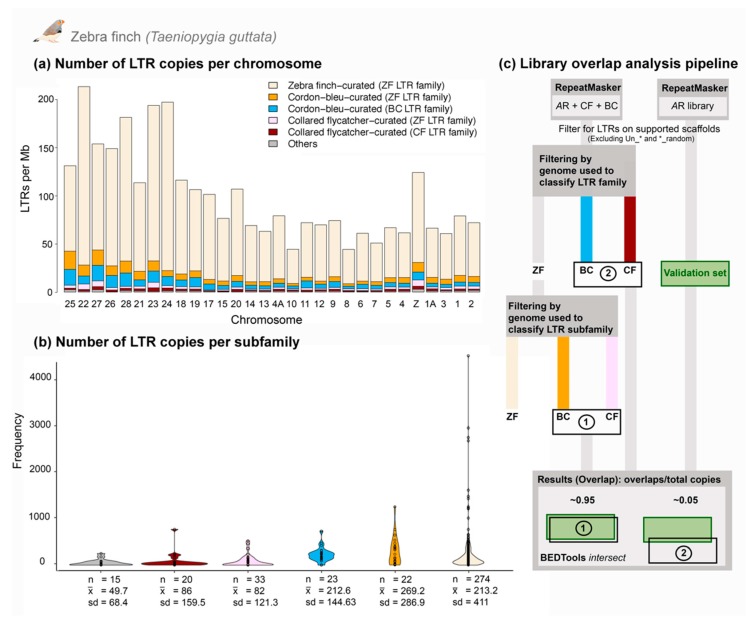
Investigation of LTR subfamily number and diversity in the zebra finch genome. We masked the genome of zebra finch (taeGut2) using RepeatMasker and two repeat libraries. One consisted of *Aves* repeats in Repbase (*A*R) only, and the other contained Repbase repeats with addition of collared flycatcher [22] and the novel blue-capped cordon-bleu repeats. We found ~4.5 Mb more LTR elements using the latter library (Table S4). Panel (**a**) shows the number of LTRs per Mb per chromosome. LTR copies were grouped according to the genome assembly used for curation and species first used for LTR family definition. Chromosomes are ordered in ascending size and are named according to homology with chicken chromosomes. Panel (**b**) shows the number of LTR copies per subfamily, here depicted as violin distributions. Statistics presented for each group of LTR copies per subfamily are: sample size per category (n), mean ($\bar{x}$) and standard deviation (sd) of copies per subfamily per category. Panel (**c**) shows the library overlap analysis pipeline. Several steps are shared with the other analyses depicted in (**a**) and (**b**). Blue-capped cordon-bleu (BC) and collared flycatcher (CF) LTRs belonging to zebra finch (ZF) LTR families generally map to already annotated repeats (overlaps/total copies ≈ 0.95). LTR copies from families described as novel in respective genome project map to new positions (overlaps/total copies ≈ 0.05).

**Figure 5:**
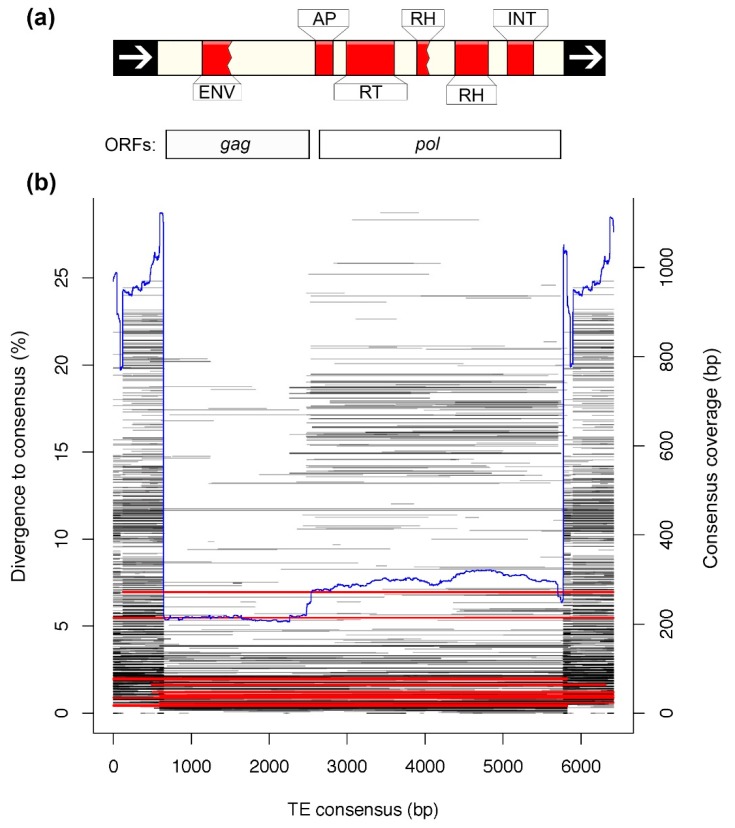
Functional domain annotation and genomic BLASTn hits of TguERVL2_I_Ucy. We predicted conserved domains and open reading frames (ORFs) of the consensus sequence of TguERVL2_I_Ucy (**a**). In addition to the canonical domains (AP, RT, two RH (one partial and one complete), and INT), a disrupted ENV domain was predicted at an upstream position. Panel (**b**) shows the distribution of copies of a pseudo full-length ERV consensus sequence (same LTR flanked by separately classified internal portion) of the TE subfamily TguERVL2_I_Ucy in blue-capped cordon bleu. Most copies in the genome are solo-LTRs and a majority of the full-length copies are less than 5% diverged from the consensus sequence. Hits spanning a majority of the consensus are shown in red and partial hits are black. Blue line represents consensus coverage.

We created TE landscape plots using the .align files of the RepeatMasker output as described in preceding publications [22,48], except that CpG sites have lower weighting instead of being excluded when counting substitutions (Figure 2). Data presented in Table 2 were obtained from the .tbl file of the RepeatMasker output. We investigated the respective amount of shared and lineage-specific diversity of LTR families and subfamilies using genomes and LTR libraries from in-depth curated birds: chicken—galGal4, zebra finch—taeGut2, collared flycatcher—ficAlb1.5 and blue-capped cordon-bleu, using reciprocal BLASTn searches (E-value cutoff = 10^−10^) [22] (Figure 3). The zebra finch genome (taeGut2) was masked with two libraries: a library consisting of repeats from the *Aves* category in Repbase and a “full” library where blue-capped cordon-bleu and collared flycatcher repeats were added (Table S4). Statistical analyses of chromosomal content and LTR subfamily number in zebra finch were performed using R ver. 3.5 [49] on a taeGut2 genome assembly acquired from UCSC [50] (Figure 4). Scaffolds with Un* prefix and *random suffix were excluded in the analyses (Figure 4). All repeat libraries were obtained from Repbase [43] except for the collared flycatcher library which was acquired from dfam_consensus [51]. We hypothesized that LTR subfamilies from blue-capped cordon-bleu and collared flycatcher that are more similar to zebra finch LTRs should compete more in masking with zebra finch LTRs in RepeatMasker. Conversely, we predicted that blue-capped cordon-bleu and collared-flycatcher LTR subfamilies that do not belong to a family curated in zebra finch should contribute more to the discovery of previously unannotated repeats in the taeGut2 genome. We tested this prediction by comparing the overlap of chromosomal positions between LTRs from the RepeatMasker output of the *Aves* Repbase library and two sets of LTRs from the output when masking with the “full” library, using the intersect utility in the BEDTools suite [52] (Figure 4c). To annotate a single internal portion of an ERV-like element, we reran the pipeline described above for collecting BLASTn hits along with flanking regions, to obtain more copies of the internal element. We then used the NCBI ORFfinder tool to identify open reading frames [53], NCBI CD-search for characterization of conserved domains [54], and the consensus2genome R script [55] to depict genomic hits (BLASTn) of a concatenated consensus sequence of the ERV internal region and the flanking LTRs (Figure 5).

### 2.4. Data Deposition

Linked-read data were deposited in Sequence Read Archive (accession number SRR8873500). Both the “pseudohaploid” genome assembly draft and a phased diploid assembly draft were deposited in Dryad (http://dx.doi.org/10.5061/dryad.322gd5p). The newly curated consensus sequences were deposited in dfam_consensus. 

## 3. Results

### 3.1. Genome Assembly and Karyotype of Blue-Capped Cordon-Bleu

We sequenced the genome of a male blue-capped cordon-bleu using the 10X Genomics Chromium linked-read platform [24] and obtained an average molecule length of 42.4 kb (Table 1). We assembled the genome using Supernova 2.0 and obtained an ~1.1 Gb assembly size, of which 105.6 Mb are “N” gaps, a scaffold N50 of 10.9 Mb, and a contig N50 of 66.3 kb (Table 1). We assessed the completeness of the genome using the aves_odb9 ortholog data set in BUSCO and recovered 90.1% of the genes completely, while 5.9% were fragmented and 4% were missing (Table 1). 

Next, we karyotyped male and female blue-capped cordon-bleu using Giemsa staining and C-banding (Figure 1). Like zebra finch, blue-capped cordon-bleu has 2n = 80 [32]. Unlike zebra finch where the W is smaller than the Z [32,56], blue-capped cordon-bleu has sex chromosomes of roughly equal size (Figure 1a). Sex chromosomes were identified as a homomorphic macrochromosome pair in males (ZZ), while in females they were heteromorphic (ZW). Giemsa staining pattern is shown for the largest macrochromosomes and sex chromosomes of a female (Figure 1a). C-banding revealed a highly heterochromatic W chromosome, further confirming its identity (Figure 1b). Constitutive heterochromatin on autosomes is mainly restricted to putatively centromeric regions (Figure 1b).

### 3.2. The Transposable Element Landscape of Blue-Capped Cordon-Bleu

We identified transposable elements in the genome of blue-capped cordon-bleu using de-novo prediction with RepeatModeler followed by manual curation of all non-redundant and curatable consensus sequences. Masking the genome with RepeatMasker revealed a TE content of 6.44% (Table 2), a number typical for birds [2]. Most transposons were LINEs (132,734 copies) followed by LTR retrotransposons (61,457 copies). However, they have a roughly similar density, indicating that LTR retrotransposons are longer on average (Table 2). In Figure 2, we show three TE landscapes to highlight the difference in results when only relying on previously annotated TEs (Figure 2a), adding a RepeatModeler library (Figure 2b) and when performing in-depth manual curation (Figure 2c). Many repeats were initially classified as unknown by RepeatModeler (compare Figure 2a,b). Our manual curation showed that all curatable “unknown repeats” were in fact solo-LTRs of ERV-like retrotransposons (Table S1, Figure 2c). We used the canonical 5′-TG…CA-3′-ends and TSDs to identify solo-LTR elements. However, several variations deviating from 5′-TG…CA-3′ were observed (Table S1). Following previous LTR annotations for songbirds [15,20], we classified LTR elements to ERV superfamilies based on the length of their TSDs [41]. A peculiar element—*UcyLTR-Lurtz*—had both 5 and 6 bp target site duplications. In total, 25 new families and 50 new subfamilies of retrovirus-like LTR retrotransposons were curated. Moreover, we identified 16 new CR1 subfamilies and one new CR1-mobilized tRNA-Ile SINE subfamily (Table S1, Figure S1). We found no new curatable DNA transposons, which is perhaps not surprising considering that previous investigations of estrildid finches revealed only a relatively old hAT DNA transposon family, present in low copy numbers in zebra finch [17].

### 3.3. Comparative Genomics Revealed Extensive Shared Diversity of LTRs among Estrildid Finches

From the 50 discovered ERV-like LTR retrotransposons in blue-capped cordon-bleu, we classified 25 as new families based on the lack of extensive nucleotide similarity to LTR elements in Repbase, in collared flycatcher, and to each other. We considered consensus sequences with less than 95% nucleotide identity to each other as separate subfamilies within such a family. To investigate the amount shared LTR diversity between the in-depth curated birds (chicken, collared flycatcher, zebra finch and blue-capped cordon-bleu), we extended the reciprocal BLASTn search of Suh et al. [22] using consensus sequences from blue-capped cordon-bleu. In brief, separate libraries of LTR subfamily consensus sequences from each species were BLASTn searched to each genome, and the presence and absence of LTR families and subfamilies was scored (Tables S2 and S3 and Figure 3). A majority of LTR subfamilies that was curated using the blue-capped cordon-bleu genome is shared between zebra finch and blue-capped cordon-bleu. Thus, 21 of 25 novel ERV-like LTR families are present in the zebra finch genome assembly (taeGut2) but were previously undiscovered. Four families (UcyLTRK7, UcyLTRK15, UcyLTRL6, and UcyLTR-Lurtz) are lineage-specific to blue-capped cordon-bleu (Figure 3). Only TguERV5 is specific to zebra finch (Figure 3).

To understand how heterospecific TE libraries can improve repeat annotation in a model organism and why substantial LTR diversity was previously undetected in the zebra finch, we masked the zebra finch reference genome (taeGut2, based on same isolate as taeGut1) obtained from UCSC [50], using RepeatMasker and two libraries. One library consisted of *Aves* repeats in Repbase only and the other was *Aves* Repbase repeats concatenated with collared flycatcher and blue-capped cordon-bleu repeats. The latter, “full” library masked ~7.5 Mb more repeats than the former, of which ~4.5 Mb are LTR elements and ~2.6 Mb are satellite DNA (Table S4). We visualized the chromosomal content of LTR elements by six different categories and grouped them according to two criteria: (1) songbird species whose genome assembly was used for curation and (2) whether or not the LTR element belongs to a zebra finch LTR family (Figure 4a). One exception is TguLTRL3-L_Ucy, which fulfilled our criteria to be classified as a new family but was highly similar (>75%) at two different parts of its consensus to TguLTRL3. We therefore treated this new family as belonging to the category of zebra finch (ZF) families in these analyses. One LTR subfamily (fAlbLTR1_Ucy) in blue-capped cordon-bleu (BC) belonged to a collared flycatcher (CF) LTR family and was categorized as “Others” along with mostly chicken LTRs. Note that LTR annotation by RepeatMasker includes fragments of elements, which we included in the copy number estimates. Furthermore, five BC and two CF LTR subfamilies curated using respective genome had less than five hits in total and their presence/absence in the ZF genome should thus be considered with caution. The reciprocal BLAST approach should give a more conservative picture of the genomic presence/absence status of specific LTR families and subfamilies (Figure 3). 

We observed that BC LTRs were overall more frequent than CF LTRs in the zebra finch genome (Figure 4a). The same pattern was seen for the frequency of LTR copies per subfamily, with a total of 10,811 BC copies and 4427 CF copies (Figure 4b). There were significantly more BC LTR copies per subfamily than CF copies (Welch *t*-test; *p*-value = 1.151 × 10^−4^). We saw the same trend when we compared LTR subfamilies from BC and CF families, with 4889 copies from BC LTR families and 1719 copies from CF families, and significantly more BC than CF copies per LTR subfamily (Welch *t*-test; *p*-value = 9.949 × 10^−3^). Furthermore, BC LTRs from BC families constituted significantly more base pairs per chromosome than CF LTRs from CF families (Wilcoxon signed rank test; *p*-value = 1.863 × 10^−9^). BC LTRs from BC families comprised in total 1736 kb compared to 471 kb of CF LTRs from CF families. These results strongly indicate that in-depth curation of LTR families in the more closely related blue-capped cordon-bleu led to annotation of more LTR copies in zebra finch than did the LTR families of the more distantly related collared flycatcher.

Long terminal repeat subfamilies from BC and CF belonging to ZF LTR families have high sequence similarity to zebra finch LTRs and would therefore compete in masking with them. We can call this the “competition-in-masking” hypothesis. A prediction from this hypothesis is that the largest gain in finding previously unannotated LTR elements in zebra finch should be obtained by using consensus sequences from LTR families previously undetected in zebra finch. We tested the “competition-in-masking” hypothesis by counting the number of overlaps between LTRs from the RepeatMasker output using only *Aves* Repbase repeats as library against two sets of LTRs from the “full” library (Figure 4c). The first set consisted of BC and CF LTRs belonging to ZF LTR families (8651 copies), and the second set consisted of BC and CF LTRs belonging to respective BC or CF LTR families (6608 copies). In the first set, 8214 overlaps were found which gave an overlap/copy number ratio of ~0.95. In the second set, only 373 overlaps were counted which results in a ratio of ~0.05. These results strongly confirm the “competition-in-masking” hypothesis and highlight how describing novel LTR families in a non-model relative can uncover hidden LTR diversity in the genome of a model organism.

### 3.4. Analysis of a Recently Active TE

We were able to curate a full-length LTR retrotransposon subfamily from the ERVL superfamily with complete internal region, in the blue-capped cordon-bleu genome. The copies of this LTR subfamily, TguERVL2_I_Ucy, make up ~1 Mb in total, which is 2.5 times more DNA than the closely related TguERVL2_I in the zebra finch genome (Table S5). The low average divergence (1.7%) to the consensus sequence is a good indication that this TE subfamily was very recently active (Table S5). We did a functional annotation of the consensus sequence of TguERVL2_I_Ucy, which revealed two long ORFs in the same reading frame and intact AP, RT, RH and INT domains, as well as an additional broken RH domain, all of which are canonical for vertebrate ERV-like retrotransposons (Figure 5a) [44,59,60]. However, the AP domain is predicted partially outside of the ORF boundaries (Figure 5a). Curiously, a disrupted envelope (ENV) glycoprotein C domain from the Marek_A superfamily is predicted inside the *gag* ORF (137 amino acid alignment to superfamily member PHA03269, E-value = 7.97 × 10^−4^). The Marek_A glycoprotein was originally classified in Marek’s disease virus (also known as *Gallid alphaherpesvirus*) [61], a ~174–180 kb dsDNA herpesvirus causing a neoplastic disease in chickens [62,63]. Interestingly, the TguERVL2 family is found in chickens as well (Table S3).

Conserved domain analysis of TguERVL2_I and TguERVL1_I in zebra finch suggests that these have all domains except for this broken ENV (not shown). However, a protein alignment of consensus sequences of TguERVL2_I, TguERVL1_I and members of PHA03269 (Envelope glycoprotein C from *Human alphaherpesvirus 3* and *Cercopithecine alphaherpesvirus 9*) and pfam02124 (various herpesviruses) revealed that all TguERVL2_I subfamilies, but not TguERVL1_I, share similarity in a short region mainly to PHA03269 (Figure S2; Data S2). It is therefore likely that this hit represents an ancestral feature of the TguERVL2 family and not a translocation or recombination with a herpesvirus in the recent history of blue-capped cordon-bleu. Furthermore, this amino acid feature does not mean that TguERVL2_I_Ucy has an intact envelope as has been seen for some invertebrate LTR retrotransposons that likely acquired an entire ENV ORF from dsDNA viruses [64].

We inferred a maximum likelihood phylogeny of internal consensus sequences of TguERVL2_I in blue-capped cordon-bleu, zebra finch and collared flycatcher to analyze the evolutionary history of this LTR family (Figure S3). TguERVL1_I was the most closely related TE in the well-annotated zebra finch genome and was consequently chosen as outgroup. The phylogeny recapitulated the species tree with strong support (99 of 100 bootstrap replicates), indicating that TguERVL2_I has been vertically inherited in the investigated songbirds. 

The curated LTR of TguERVL2_I_Ucy was concatenated with both ends of the internal region to create a 6.4 kb “pseudo full-length” ERV consensus, which we subsequently used to characterize consensus coverage of hits in the genome using the consensus2genome R script relying on BLASTn (E-value cutoff = 10^−7^) [55]. Most copies of this ERV throughout the genome are solo-LTRs, as indicated by the higher coverage of terminal repeats (Figure 5b). We also see a pattern of more copies with intact internal regions being recently diverged from the consensus (Figure 5b). These observations are consistent with the view of deletion of the internal region and one LTR, through within-element non-allelic homologous recombination [65]. Curiously, many hits in the range of 10% to 20% divergence to consensus seem to lack homology for the first ORF containing the broken ENV. These likely represent elements of another LTR subfamily with a similar *pol* ORF but a dissimilar *gag* ORF. 

## 4. Discussion

In this study, we present the third linked-read genome assembly of any bird, to our knowledge. If we compare with one of the 10X genome assemblies published previously for eastern black-eared wheatear (*Oenanthe hispanica melanoleuca*) [66], we obtain a higher weighted average molecule length (42.4 kb vs. 17.5 kb) which most likely contributes to a higher scaffold N50 (10.9 Mb vs. 90 kb), (Table 1). Even higher scaffold N50 may be possible to be obtained by using a subset of reads [23], as shown by Toomey et al. [67] who produced a 10X genome assembly with scaffold N50 of 18.97 Mb for Gouldian finch (*Erythrura gouldiae*) with a read-depth of 60-fold in Supernova. Using the ranking employed by Suh and Kapusta [2], the genome assembly of blue-capped cordon-bleu is of medium quality (scaffold N50 >1 Mb; high quality requiring chromosome-level scaffolds) and has the 11th highest scaffold N50 out of the 77 analyzed bird genomes [2]. We also present the fourth bird genome with a well-curated transposon library and the first that allows comparative TE biology on the within-family level in birds. Previous work has shown that zebra finch has a substantial recent accumulation of ERV-like retrotransposons compared with other bird lineages [2,17,22], but see Mason et al. for a different view of LTR retrotransposon abundance in chickens [68]. The genome of blue-capped cordon-bleu also shows ERV-like LTR retrotransposon accumulation, and notably a recent expansion mostly caused by a single LTR subfamily, TguERVL2_I_Ucy (Table S5). Considering that zebra finch and blue-capped cordon-bleu separated at the deepest node of Estrildidae 10 Mya [15,69], ERV-like LTR retrotransposon accumulation might be ancestral to this clade. 

Curiously, a majority (21 out of 25) of ERV-like LTR families described in this study are shared with zebra finch but were not previously described in its repeat annotation [17]. By combining repeats curated from the closely related blue-capped cordon-bleu (BC) and the more distantly related collared flycatcher (CF) with the *Aves* Repbase library, we were able to mask an additional 4.5 Mb (>10% increase) LTR retrotransposons in the zebra finch genome. We found significantly more copies per subfamily and a larger number of base pairs masked per chromosome of BC LTRs from BC families than CF LTRs from CF families (Figure 4a,b). This indicates that phylogenetic relatedness is an important factor when trying to find more repeats in a genome assembly using a TE library from another species. Furthermore, by analyzing the overlap between LTR copies in the RepeatMasker output from of the *Aves* Repbase and “full” libraries, we see that the largest addition of previously unannotated LTR elements in the taeGut2 genome results from novel BC and CF LTR families (Figure 4c). These results indicate that there are more TEs to be found in the reference genomes of model organisms and that they may be discovered by curating the repeatomes of closely related species.

A few novel BC ERV-like LTR retrotransposon families do not occur in zebra finch (Figure 3). Some or all might be unassembled or lost by drift or selection in zebra finch. A more plausible explanation is that they constitute recent germline infiltrations in the blue-capped cordon-bleu lineage. If that was the case for all four novel ERV-like LTR retrotransposon families, then the rate of germline infiltration in the blue-capped cordon-bleu lineage would be one every 2.5 My. This number may be an underestimate considering that research on a recent germline infiltration in koalas (*Phascolarctos cinereus*) show a polymorphic presence/absence pattern and no fixed insertions among individuals [70,71,72,73]. In addition, note the 28 LTR families on the short branch shared by zebra finch and blue-capped cordon-bleu (Figure 3). This indicates an even higher rate of germline infiltrations in the common ancestor of estrildid finches. The results presented here give an indication that the repetitive content and diversity of avian genomes may currently be somewhat underestimated. It is likely that the we will see diminishing returns in finding further shared TE diversity as more species are investigated. However, in-depth curation may greatly improve the accuracy of inferring a genome’s repeat landscape, especially when in-depth TE libraries from closely related species are missing [74], or when many solo-LTRs are automatically classified as unknown by RepeatModeler as was the case here for blue-capped cordon-bleu (Figure 2b).

In this particular case, variation among species in the effect of sequence modification by TE suppression systems may be increasing LTR sequence diversity in estrildid finches compared with other songbird lineages. It has previously been shown that zebra finch LTR retrotransposons frequently are C→U-modified by APOBEC family proteins leading to a G→A mutation on the antisense strand [75]. Among 111 analyzed vertebrates, APOBEC modification was especially strong in zebra finch [75] and we speculate that it could be one of the most important drivers increasing the genetic diversity of LTR subfamilies in blue-capped cordon-bleu as well as zebra finch. Knisbacher and Levanon [75] observed a much more limited effect of APOBEC in medium ground finch (*Geospiza fortis*) indicating that APOBEC activity varies among songbirds or that the edited sites were more easily detected in zebra finch because of its in-depth curated LTR library. However, it is possible that APOBEC modification mainly affects LTR subfamily diversity, see for example the high number of subfamilies on the zebra finch branch in Figure 3. On the other hand, LTR families with no homology to other repeats in Repbase likely represent previously undiscovered retroviral diversity arising from germline infiltrations. Altogether, genome evolution in Estrildidae may very well be shaped by the expansion of LTR retrotransposons and their strong suppression by APOBEC modification. 

The question of shared ERV-like retrotransposon diversity warrants further study, both in Estrildidae and in other songbird clades. Related to the question of shared diversity is the notion that a single LTR subfamily, TguERVL2_I_Ucy, has proliferated very recently in the evolutionary history of the blue-capped cordon-bleu so that it now composes 2.5-fold more DNA than in the genome of its closest relative in zebra finch (Table S5). This number is probably an underestimate considering the difficulty in assembling long repeat sequences with high sequence identity [76]. The fact that a full-length element of 6.4 kb was curatable and the consensus has intact GAG, AP, RT and RH domains suggests that this subfamily is likely still actively retrotransposing. The phylogeny of TguERVL2_I_Ucy and its closest songbird relatives suggests vertical inheritance of this LTR family at least since the common ancestor of Estrildidae (Figure S3). The ultimate cause of this element’s high frequency in blue-capped cordon-bleu could be random genetic drift or some molecular feature of its Gag polyprotein—such as the putative Envelope glycoprotein C domain—that has allowed it to escape effective suppression. A horizontal acquisition event may have occurred in either direction between the ancestor of TguERVL2 and an alphaherpesvirus, but we cannot rule out that the similarity to Envelope glycoprotein C is caused by genetic drift or adaptive molecular convergence alone. However, horizontal transfer in both directions between LTR retrotransposons and dsDNA viruses have previously been inferred, which implies that such events do occur successfully [64,77].

Further investigation in Estrildidae is needed to explore the link between ERV-like LTR retrotransposon activity and the high rate of chromosomal inversions observed in this songbird clade [15]. For example, a single insertion of an LTR retrotransposon, *Ty912*, has been shown to increase the rate of gross chromosomal rearrangements (such as inversions) 380-fold in an experimental *Saccharomyces cerevisiae* yeast strain, compared to a wild type strain [12]. The karyotype data we present here indicates that no major interchromosomal rearrangements (i.e., fissions or fusions) have occurred since the divergence of zebra finch and blue-capped cordon-bleu (Figure 1) [32]. Future studies would do service by comparing the number of intrachromosomal rearrangements (especially inversions) in Estrildidae with other bird clades and investigate their likely link with LTR retrotransposon proliferation. 

To conclude, we were able to annotate an additional 4.5 Mb of LTR retrotransposons in zebra finch using the in-depth curated LTR libraries of collared flycatcher and, most importantly, blue-capped cordon-bleu. We were also able to uncover a shared estrildid diversity of 21 out of 25 previously undiscovered ERV-like retrotransposon families found in blue-capped cordon-bleu. These results demonstrate the significance of studying close relatives to model organisms. 

## Figures and Tables

**Table 1:** Sequencing and assembly statistics for the genome assembly of blue-capped cordon-bleu.

Statistic	Quantity
Assembly size	1099.6 Mb
“N” nucleotides	105.6 Mb
Weighted mean molecule length	42.4 kb
Number of reads	254.2 million
Scaffolds	26,389
Scaffold N50	10.9 Mb
Contigs	51,469
Contig N50	66.3 kb
BUSCO (complete)	90.1%
BUSCO (fragmented)	5.9%
BUSCO (missing)	4%

**Table 2:** Copy number, total base pair and density of different classes of repetitive elements annotated by RepeatMasker using a library consisting of manually curated blue-capped cordon-bleu and collared flycatcher repeats, and the *Aves* library from Repbase.

Repeat Type	Copies	Total bp	% of Genome
SINE	7163	852,236	0.08
LINE	132,734	37,876,706	3.44
LTR	61,457	29,437,443	2.68
DNA	14,100	2,195,734	0.20
Unclassified	2367	416,198	0.04
Total interspersed repeats	217,821	70,778,317	6.44
Small RNA	1479	199,270	0.02
Satellites	1960	581,825	0.05
Simple repeats	211,440	9,408,016	0.86
Low complexity	43,325	2,238,772	0.20
Total tandem repeats	258,204	12,427,883	1.17
Total repeats	746,059	83,206,200	7.61

## Supplementary Materials

The following are available online at https://www.mdpi.com/2073-4425/10/4/301/s1, Figure S1: Maximum likelihood phylogeny of CR1 consensuses; Figure S2: Snapshot of protein alignment of TguERVL_I Gag and Envelope glycoprotein C members (PHA03269 and pfam02124) of Marek_A superfamily of alphaherpesviruses; Figure S3: Maximum likelihood phylogeny of songbird TguERVL2_I consensuses; Table S1: Classification sheet of de-novo curated repetitive elements; Table S2: LTR reciprocal BLAST among in-depth curated birds, LTR subfamilies per branch; Table S3: LTR reciprocal BLAST among in-depth curated birds, LTR families per branch; Table S4: Comparison of RepeatMasker output between two different libraries (*Aves* Repbase library vs. “full” merged library) when masking zebra finch; Table S5: Abundance of TguERVL2_I family among in-depth curated songbirds; Data S1: Fasta-formatted consensus sequences of blue-capped cordon-bleu TEs; Data S2: Protein alignment of TguERVL_I Gag and Envelope glycoprotein C members (PHA03269 and pfam02124) of Marek_A superfamily.
