# Supplementary material for: The Genome of Blue-Capped Cordon-Bleu Uncovers Hidden Diversity of LTR Retrotransposons in Zebra Finch
Source: Genes (Basel). 2019 Apr 13;10(4):301. doi: 10.3390/genes10040301 (PMC6523648; doi:10.3390/genes10040301)
Supplement: Supplementary file 1 [file genes-10-00301-s001.zip › uraCya_genome_paper_SUPPLEMENT_proofs.docx]

**Figure S1.** Maximum likelihood phylogeny of CR1 consensuses. PhyML phylogeny of novel CR1 elements from blue-capped cordon-bleu (blue) and the same zebra finch (black), chicken (grey) and collared flycatcher (red) CR1 subfamilies used in Suh *et al.* (2018). Values on branches are bootstrap support values (100 replicates). The tree is rooted using PSLINE (green) from turtles.

**
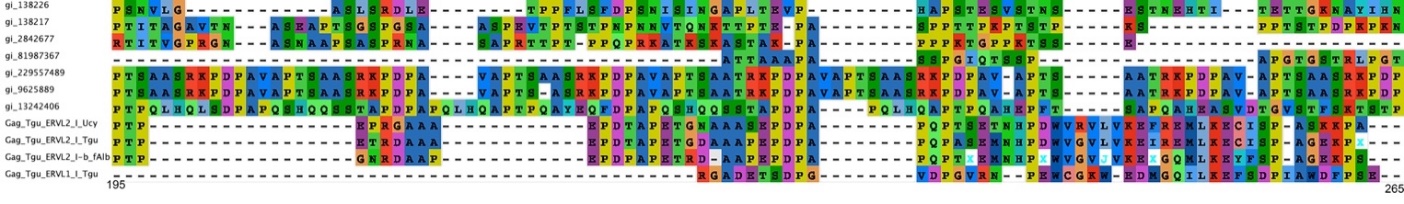
**

**Figure S2.** Snapshot of protein alignment of TguERVL_I Gag and Envelope glycoprotein C members (PHA03269 and pfam02124) of Marek_A superfamily of alphaherpesviruses. There are some similarities in the depicted region (position 195-265 in alignment) between the envelope glycoproteins and ORF1 of TguERVL2 LTR retrotransposons. Overall, the LTR retrotransposons and Envelope glycoprotein C sequences cluster separately.

**
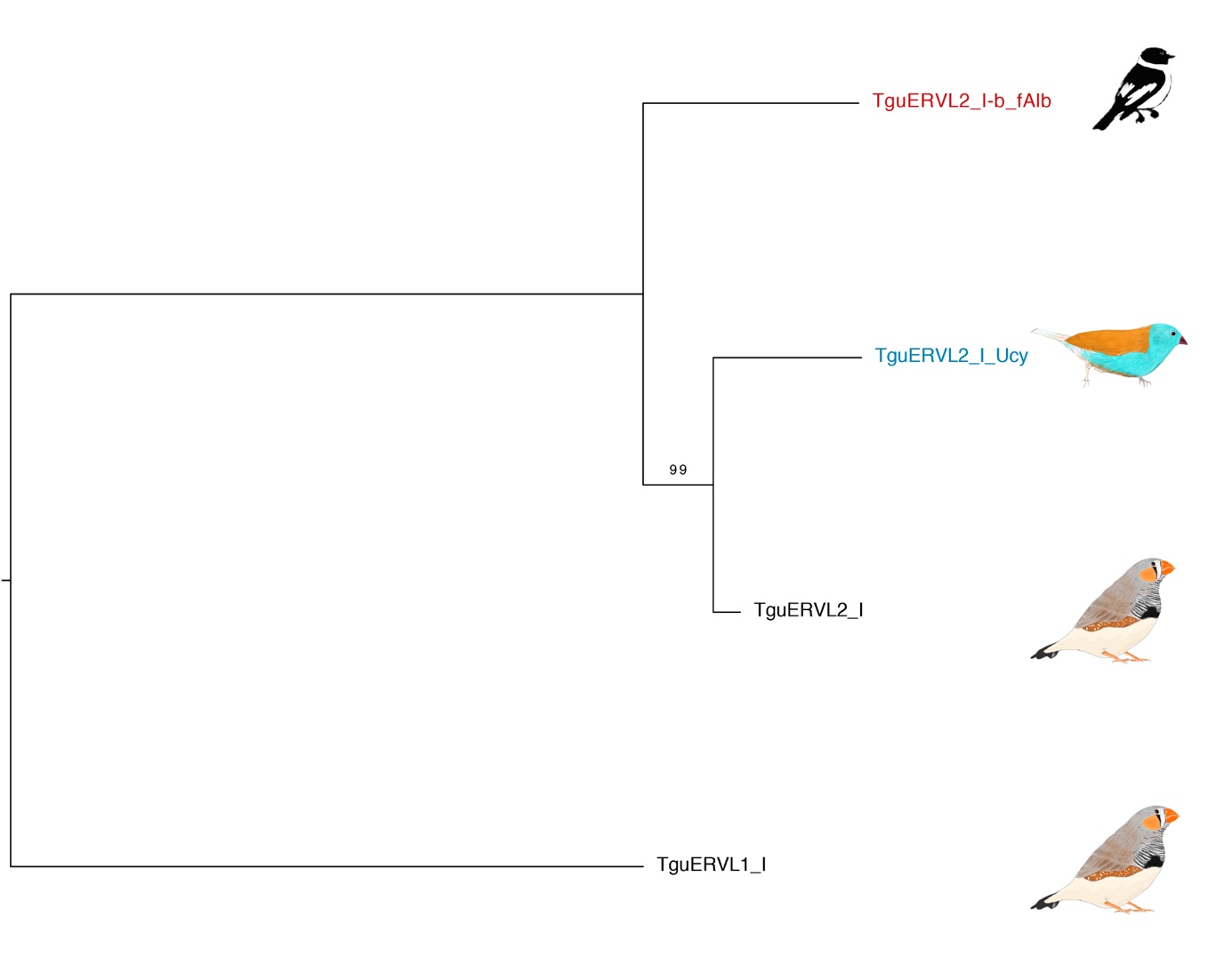
**

**Figure S3.** Maximum likelihood phylogeny of songbird TguERVL2_I consensuses. A PhyML phylogeny of the internal portion of the endogenous retrovirus family TguERVL2 and its closest relative in zebra-finch, TguERVL1. Due to its short consensus length, TguERVL2_I-a_fAlb was not included. The phylogeny recapitulates the species tree, suggesting vertical inheritance of this ERV family. Values on branches are bootstrap support values (100 replicates).

**Table S1.** Classification sheet of de-novo curated transposable elements (provided in a

separate file).

**Table S2.** LTR reciprocal BLAST among in-depth curated birds, LTR subfamilies per branch (provided in a separate file).

**Table S3.** LTR reciprocal BLAST among in-depth curated birds, LTR families per branch (provided in a separate file).

**Table S4.** Comparison of RepeatMasker output between two different libraries (*Aves* Repbase vs. “full” merged library) when masking zebra finch. We compared the RepeatMasker (.tbl file) output when masking the taeGut2 zebra finch genome using two different libraries: one with only *Aves* repeats and another (full library) with *Aves*, collared flycatcher and blue-capped cordon-bleu repeats. The latter masks ~7.5 Mb more repeats amounting to ~7.2 % more bases (0.49 more percentage points).

|  |  | **taeGut2 *Aves* lib.** |  |  | **taeGut2 full lib.** |  |
| --- | --- | --- | --- | --- | --- | --- |
| **Repeat type** | **Copies** | **Total bp** | **Total %** | **Copies** | **Total bp** | **Total %** |
| SINE | 8,016 | 936,744 | 0.08 | 8,110 | 943,449 | 0.08 |
| LINE | 147,468 | 42,552,906 | 3.45 | 154,021 | 43,056,740 | 3.49 |
| LTR | 80,571 | 42,368,712 | 3.44 | 93,697 | 46,846,109 | 3.80 |
| DNA | 15,565 | 2,412,384 | 0.20 | 15,564 | 2,412,317 | 0.20 |
| Unclassified | 2652 | 462,702 | 0.04 | 2,651 | 462,576 | 0.04 |
| Total interspersed repeats | 254,272 | 88,733,448 | 7.20 | 274,043 | 93,721,191 | 7.61 |
| Small RNA | 1,875 | 235,684 | 0.02 | 1864 | 235,074 | 0.02 |
| Satellites | 989 | 111,657 | 0.01 | 4506 | 2,722,457 | 0.22 |
| Simple repeats | 279,369 | 12,623,464 | 1.02 | 278118 | 12,565,128 | 1.02 |
| Low complexity | 55,610 | 2,900,592 | 0.24 | 55250 | 2,876,967 | 0.23 |
| Total tandem repeats | 337,843 | 15,871,397 | 1.29 | 339,738 | 18,399,626 | 1.49 |
| Total repeats | 529,115 | 104,604,845 | 8.49 | 613781 | 112,120,817 | 9.08 |

**Table S5.** Abundance of TguERVL2_I family among in-depth curated songbirds. Comparison of the internal ORF-containing portion of TE family TguERVL2 in zebra finch, blue-capped cordon bleu, and collared flycatcher.

| **TE subfamily** | **Species (genome)** | **Library ^1^ used for RepeatMasker** | **Size of all copies (percentage of genome)** | **Weighted average divergence from consensus (Kimura-2-Parameter)** |
| --- | --- | --- | --- | --- |
| TguERVL2_I | *Taeniopygia guttata* | *A*R | 413.4 kb (0.03 %) | 18.95 |
| TguERVL2_I | *Uraeginthus cyanocephalus* | *A*R+CF+BC | 257 kb (0.02%) | 19.17 |
| TguERVL2_I_Ucy | *Uraeginthus cyanocephalus* | *A*R+CF+BC | 1034 kb (0.09 %) | 1.70 |
| TguERVL2_I-b_fAlb | *Uraeginthus cyanocephalus* | *A*R+CF+BC | 21 kb |  |
| TguERVL2_I | *Ficedula albicollis* | *A*R+CF | 310 kb (0.03%) | 18.85 |
| TguERVL2_I-a_fAlb | *Ficedula albicollis* | *A*R+CF | 105 kb (0.01%) | 20.83 |
| TguERVL2_I-b_fAlb | *Ficedula albicollis* | *A*R+CF | 156 kb (0.01%) | 13.71 |

^1^ *A*R = Aves Repbase, CF = Repeat library from curation in collared flycatcher, BC = Repeat library presented here for blue-capped cordon-bleu.

**Data S1.** Fasta-formatted consensus sequences of blue-capped cordon-bleu TEs.

**Data S2.** Protein alignment of TguERVL_I Gag and Envelope glycoprotein C members (PHA03269 and pfam02124) of Marek_A superfamily.
